# Supplementary material for: Unraveling the Role of Hepatic PGC1α in Breast Cancer Invasion: A New Target for Therapeutic Intervention?
Source: Cells. 2023 Sep 19;12(18):2311. doi: 10.3390/cells12182311 (PMC10529029; doi:10.3390/cells12182311)
Supplement: Supplementary file 1 [file cells-12-02311-s001.zip › cells-2580099-supplementary.pdf]

# Unraveling the Role of Hepatic PGC1α in Breast Cancer Invasion: A New Target for Therapeutic Intervention?

**Kumar Ganesan[1], Cong Xu[1], Qingqing Liu[1], Yue Sui[1], Jianping Chen[1,2*]**

[1]School of Chinese Medicine, LKS Faculty of Medicine, The University of Hong Kong, Sassoon Road, Hong Kong, China
[2]Shenzhen Institute of Research and Innovation, The University of Hong Kong, Shenzhen 518057, China
* Correspondence: abchen@hku.hk; Tel.: +86-852-39176479; Fax: +86-852-28725476

**Supplementary Table S1. Silencing of PGC1α gene in BC cells by transfection using Lipofectamine RNAiMAX**

| S.No | Gene | Sequence | |
|---|---|---|---|
| | | Sense ( 5'-3' ) | Antisense ( 5'-3' ) |
| 1 | PGC1α | GCUCGGAGCUUCUCAAAUATT | UAUUUGAGAAGCUCCGAGCTT |
| 2 | PGC1α | GGACAGUGAUUUCAGUAAUTT | AUUACUGAAAUCACUGUCCTT |
| 3 | PGC1α | GCACGCAAUCCUAUUCAUUTT | AAUGAAUAGGAUUGCGUGCTT |
| 4 | NC | UUCUCCGAACGUGUCACGUTT | ACGUGACACGUUCGGAGAATT |

**Supplementary Table S2. List of antibodies used in the study**

| Antibodies | Catalog number | Source | Dilution | Company Name |
|---|---|---|---|---|
| Primary antibodies | | | | |
| ACC | Cat# A15606 | Rabbit | 1:500 | Abclonal |
| Akt | Cat# A18120 | Rabbit | 1:200 | Abclonal |
| BAX | Cat# A19684 | Rabbit | 1:200 | Abclonal |
| Bcl2 | Cat# A19693 | Rabbit | 1:200 | Abclonal |
| Casp3 | Cat# A17900 | Mouse | 1:200 | Abclonal |
| Casp9 | Cat# A11910 | Rabbit | 1:200 | Abclonal |
| Cleaved Casp 3 | Cat# A22869 | Rabbit | 1:200 | Abclonal |
| COX-2 | Cat# A1253 | Rabbit | 1:1000 | Abclonal |
| CPT1 | Cat# A5307 | Rabbit | 1:500 | Abclonal |
| Cyt C | Cat# A13430 | Rabbit | 1:200 | Abclonal |
| E-cad | Cat# A18135 | Mouse | 1:200 | Abclonal |
| ERK1/2 | Cat# A10613 | Mouse | 1:200 | Abclonal |
| ERRα | Cat#A14184 | Rabbit | 1:50 | Abclonal |
| FABP-L | Cat# A11213 | Rabbit | 1:500 | Abclonal |
| FAS | Cat# A0461 | Rabbit | 1:500 | Abclonal |
| GAPDH | Cat# 2118 s | Rabbit | 1:1000 | Cell Signaling Technology |
| IL-6 | Cat# A0286 | Rabbit | 1:200 | Abclonal |
| Ki67 | Cat# A11390 | Rabbit | 1:200 | Abclonal |
| LPL | Cat# A4115 | Rabbit | 1:200 | Abclonal |

| | | | | |
|---|---|---|---|---|
| MMP2 | Cat# A6247 | Rabbit | 1:200 | Abclonal |
| MMP9 | Cat# A0289 | Rabbit | 1:200 | Abclonal |
| mTOR | Cat# A2445 | Rabbit | 1:200 | Abclonal |
| N-cad | Cat# A0433 | Rabbit | 1:200 | Abclonal |
| NFκB | Cat# A3108 | Rabbit | 1:200 | Abclonal |
| PARP1 | Cat# A19596 | Rabbit | 1:200 | Abclonal |
| PCNA | Cat# A12427 | Rabbit | 1:200 | Abclonal |
| pERK1/2 | Cat# AP0472 | Rabbit | 1:200 | Abclonal |
| PGC1α | Cat# PA572948 | Rabbit | 1:200 | Life Technologies |
| PI3K | Cat# A19742 | Rabbit | 1:200 | Abclonal |
| RAS | Cat# A19638 | Rabbit | 1:200 | Abclonal |
| SCD1 | Cat# A16429 | Rabbit | 1:200 | Abclonal |
| TNF-α | Cat# ab183218 | Rabbit | 1:200 | Abcam |
| Vimentin | Cat# A11423 | Rabbit | 1:200 | Abclonal |
| α-SMA | Cat# A17910 | Rabbit | 1:200 | Abclonal |
| β-actin | Cat# ab8226 | Mouse | 1:500 | Abcam |
| β-catenin | Cat# A11512 | Rabbit | 1:200 | Abclonal |
| Secondary antibodies | | | | |
| Mouse | Cat# 7076 | Horse | 1:500 | Cell Signaling Technology |
| Rabbit | Cat# 7074 | Goat | 1:500 | Cell Signaling Technology |
